# The Influence of Serum Uric Acid Level on Alzheimer's Disease: A Narrative Review

**DOI:** 10.1155/2021/5525710

**Published:** 2021-06-02

**Authors:** Mengyuan Qiao, Chongli Chen, Yuqing Liang, Yuxi Luo, Wenbin Wu

**Affiliations:** Department of Geriatrics, Hospital of Chengdu University of Traditional Chinese Medicine, Chengdu City, Sichuan Province, China

## Abstract

As a powerful antioxidant in the human body, uric acid (UA) has been the subject of increasing research that focused on its influence on Alzheimer's disease (AD) in recent years. The latest literature was gathered to describe the influence of serum uric acid (SUA) level on the onset and progression of AD and to analyze the possibility that SUA is a biomarker of Alzheimer's disease. A large number of existing studies suggested that the SUA level was lower or tended to decrease in patients with AD, and increased SUA level may have a protective effect in AD, which could reduce the risk of onset and slowing the course of the disease. However, some Mendelian randomization analyses suggested that genetically determined uric acid was not associated with AD risk. Existing research results are contradictory due to the high inconsistency of the studies, the selection of subjects, and other factors. UA also showed a strong association with cognitive function, and there appeared to be a gender-selective neuroprotective action. Due to its potent antioxidant properties, the low uric acid level may contribute to oxidative stress to accelerate disease progression. But some preclinical data showed a possibility that in some special cases, UA had a prooxidant properties. The possibility was raised in the discussion of the underlying mechanism that both the low uric acid level and the rapidly progressive course of the disease were the consequence of malnutrition. This paper reviews recent advances in the study of SUA and AD which offers the possibility of new biomarker, new prevention, and treatment strategies for Alzheimer's disease.

## 1. Introduction

Uric acid (UA) is a product of purine metabolism, which is a natural and powerful antioxidant that helps remove superoxide by blocking the degradation of superoxide dismutase, the enzyme responsible for purine removal [1]. The SUA level is altered according to the balance between dietary purine intake, xanthine oxidase activity, and renal UA excretion [2]. When the equilibrium is disturbed, hyperuricemia or hypouricemia occurs. In the past, uric acid was mainly considered to be related to gout. But in recent years, there has been a proliferation of studies between uric acid and neurodegenerative disease, mainly including dementia, Parkinson's disease, amyotrophic lateral sclerosis, and multiple system atrophy.

Current trends indicate that early detection of AD by noninvasive approaches is a popular area of research. A growing number of studies have demonstrated that UA was linked with the risk, progress, and prognosis of AD, through its neuroprotective effect, antioxidant capacity, metal complexation, and other mechanisms [3]. However, due to the inconsistency of research types and limitations of various research types, existing research results are contradictory. This article reviewed the progress of epidemiological and other studies on the influence of SUA on AD, aiming at exploring the possibility of SUA as a peripheral marker of AD and providing a reference for future research on peripheral markers of AD.

## 2. Method

**Figure 1:**
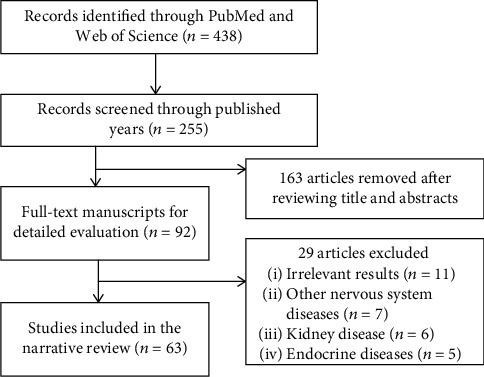
A flowchart shows the selection of study in the narrative review, which includes the final results of the initial search.

On December 04, 2020, we performed a search of PubMed and Web of Science. Language and regional restrictions were not imposed. We searched the database above using search terms including “Alzheimer” AND “uric acid”, “dementia” AND “uric acid”, and “cognitive” AND “uric acid”. The reference lists of included studies and relevant reviews were studied manually to minimize the omission of potentially eligible articles. We mainly selected literature from 2016 to 2020 and included older literature that was commonly cited and highly valued (Figure 1).

## 3. Level of SUA Was Relevant to the Risk of AD

In recent years, there has been an increasing interest in studying the risk of developing AD in populations with different concentrations of SUA. However, the relationship between level of SUA and the risk of AD has been conflicting in different types of studies, and the evidence is weak in elderly subjects.

### 3.1. Results from Epidemiological Studies

In recent years, several prospective cohort studies with large sample size and long follow-up period have been published. A prospective cohort study in Sweden enrolled a population-based sample of 1462 females who were followed for up to 44 years. During the follow-up period, serum uric acid levels were measured twice. They found that a higher SUA concentration (per standard deviation of 76.5 mmol/L) was related to a lower risk for incident dementia (*n* = 320; hazard ratio (HR) 0.81; confidence interval (CI) 0.72–0.91) and both AD (*n* = 152; HR 0.78; CI 0.66–0.91) and vascular dementia (VD; *n* = 52; HR 0.66; CI 0.47–0.94) [4]. This finding suggested the protective effect of SUA in the onset of incident dementia regardless of subtype. For the present literature, this cohort had the longest follow-up years and was a strong evidence. However, the limitation was that the sample population included only women. So whether the conclusions are also applicable to men is yet to be confirmed by other studies.

Latourte et al. evaluated the longitudinal link between the level of SUA and incident dementia in a large cohort followed for 12 years. This study enrolled 1598 individuals (mean (SD) age 72.4 (4.1) years, 61.7% female). The result showed that 110 participants developed dementia. After multivariate adjustment, the multivariate HR of the highest versus lowest SUA levels was 1.79. The correlation was stronger with other subtypes compared to AD, including VD and mixed dementia (MD) [5]. The elderly with a high level of SUA may mark the higher risk of incident dementia, especially VD or MD. The conflicting findings of these two studies may be due to the different gender structures of the participants and the duration of follow-up years.

Because of the difficulty of follow-up and other factors, only a few studies have considered the subtype classification in the cohort study of dementia. Alam et al. studied 11,169 participants in communities (ARIC) cohort with 24.1-year median follow-up period. As the study progressed, a total of 2005 cases of incident dementia were detected. They conducted adjustments including cardiovascular risk factors. After that, SUA and incident dementia showed an uncorrelated outcome (HR, 1.03; 95% CI, 0.88, 1.21). Elevated baseline levels of SUA were relevant to faster decline of cognition (25-year global *z*-score difference, -0.149; 95% CI, -0.246, -0.052) [6]. The results of this study may be due to the different influences of SUA on the onset of different subtypes of incident dementia. There was no separate subtype analysis of AD and VD in data analysis, which may lead to the cancellation of positive and negative effects, resulting in the final result of no correlation. Another prospective study conducted in the UK without subgroup analysis showed that people in the lowest level group of SUA had a 25% increased risk of dementia compared to those in the highest SUA level group (HR = 0.75, 95% CI: 0.64-0.87). SUA was an independent predictor of dementia [7]. These studies did not include the analysis of the subtypes of incident dementia. AD and VD were analyzed together, which would have some impact on the results and conclusions.

Two population-based cohort studies [8, 9] indicated that gout was inversely relevant to the onset of AD. Gout patients had lower risks of AD. Among them, Hong et al. [9] took into account the effects of vascular dementia. According to sensitivity analysis in the study, after classifying patients with stroke before AD as vascular dementia, the conclusion remained the same. However, this inverse association could not be definitively attributed to the neuroprotective effect of uric acid, since uric acid levels were not directly analyzed in these studies. It remains to be seen whether this represents a definitive causal relationship.

In addition to the cohort study, there were more retrospective and cross-sectional analysis. Boccardi et al. did a retrospective study of UA and late-onset AD. A total of 232 subjects were divided into three groups, including healthy controls (HC), mild cognitive impairment (MCI) group, and AD group (*n* = 65, 95, and 72, respectively). SUA levels were significantly lower in the AD group (4.84 ± 1.30 mg/mL) than in the healthy control group (5.82 ± 1.76 mg/mL; *P* = 0.001). After adjusting for age, sex, body mass index, and creatinine levels, the correlation that existed between SUA and AD showed that the level of SUA was independently relevant to the diagnosis of AD [10]. The results showed that AD patients had a decreased level of SUA. UA may have a protective effect on elderly AD patients. Gonzalez-Dominguez et al. investigated metabolic differences in serum of AD patients and healthy controls by using gas chromatography coupled to mass spectrometry. As a result, SUA was markedly reduced in the AD group compared to HC [11]. The same finding has been found in four investigations in recent years [12–16]. However, in a cross-sectional analysis, Zuliani et al. concluded that UA levels of LOAD (late-onset AD; 357 ± 95 *μ*mol/L) were elevated compared to the control group (300 ± 96 *μ*mol/L; *P* < 0.01) [17]. They concluded that a combination of markers including SUA may be a possible tool for the diagnosis of LOAD. In addition, three studies have reported increases or no change of UA in AD blood [18–20]. Observational evidence is susceptible to a large number of biases that limit causal inference, both residual confounding and reverse causation. It is required to conduct more studies to ascertain whether this is a causal relationship.

### 3.2. Results from Other Studies

Scholefield et al. systematically analyzed the results of various biochemical pathways in AD. The analysis showed the decrease of uric acid in biological fluids of AD cases and suggested that the decline in serum UA could be used as part of a generic biomarker for dementia [21]. In a meta-analysis, 21 case-control studies that included UA measurements were included to analyze plasma antioxidant status in AD patients and cognitively intact older adults. The result showed that plasma UA was markedly decreased in AD with a pooled mean difference (PMD) -27.37 *μ*mol/L (95% CI: -49.75, -5.00, *P* = 0.02) compared to the control group [22]. The meta-analysis of Khan et al. also showed that the level of SUA in dementia patients was markedly decreased compared to HC, especially in patients with AD [23]. Paradoxically, another analysis suggested that there was no significant difference in levels of SUA between AD patients and HC, but with proper interpretation, there may be a trend toward decreased UA in AD [24]. The above analysis suggested that high uric acid could be relevant to low risk of AD, which was also confirmed in an animal experiment. In an experimental study, Wang et al. observed differences in the expression of AD biomarkers (APP and BACE1) in rats with different serum uric acid levels. The results showed that rats in the high-SUA-level group had significantly lower protein levels of APP and BACE1 in the hippocampus [25].

Over the past few years, four two-sample Mendelian randomized analyses have been performed to investigate the relationship between genetically determined circulating UA levels and risk of AD. Mendelian randomization is a statistical method that uses genetic variants like single nucleotide polymorphisms as genetic tools to make causal inferences about the nature of exposure-outcome relationships [26]. Williams et al. extracted genotype-AD risk association statistics from data from a genome-wide association study of LOAD subjects (*n* = 17,008) and controls (*n* = 37,154) [27]. The result suggested that genetically determined UA was not associated with AD risk and higher exposure to UA does not reduce the risk of AD. Similar results could be found in the study of Efstathiadou et al. [26] and Yuan and Yang [8]. But interestingly, Wang et al. came to the opposite conclusion in the latest research in 2020. The study found that for every standard deviation, increase in levels of UA (1.33 mg/dL) was related to a 0.09-fold increase in the risk of AD [28]. This analysis suggested that genetically predicted higher level of UA may lead to a higher risk of AD. The MR method prevents bias caused by reverse causality and minimizes bias caused by measured and unmeasured confounding factors. These findings can make up for shortcomings of causality in observational studies and enrich the existing evidence.

### 3.3. Summary of This Section

Among the related studies on SUA and the risk of AD, existing cohort studies and an animal experiment suggested that high SUA level indicated a low risk of AD, and the evidence was stronger in women. Consistent with this, a large number of retrospective studies, cross-sectional analyses, and systematic meta-analyses showed that AD patients had lower SUA level than healthy controls. Conflicting conclusions of other studies may be due to the selection of subjects and the absence of subgroup analysis of dementia. Some Mendelian-random analysis indicated that gene-determined UA was not related to the risk of AD, suggesting the importance of the influence of acquired confounders.

## 4. Level of SUA Was Relevant to the Progression of AD

### 4.1. Related Studies

With the progression of AD, cognitive impairment becomes more and more serious. In recent years, only one cohort has observed cognitive decline in patients with AD at different baseline uric acid levels. Ye et al. used the AD Neuroimaging Initiative database to evaluate the cognitive decline of MCI and AD patients (*n* = 1064, including 271 HC, 596 MCI, and 197 AD). SUA and AD biomarkers in cerebrospinal fluid (CSF) were recorded once for baseline. Cognitive assessment scales were assessed continuously. The result showed that higher SUA level was related to slower cognitive decline, particularly in MCI and AD subgroups, and this association was more significant among female participants (*P* < 0.001). Cerebrospinal fluid biomarker models showed that high concentrations of UA attenuated the adverse effects of A*β*1-42 and tau on cognitive decline in female subjects. However, it was noteworthy that in male subjects, the interaction was limited to resistance to the adverse effects of A*β*1-42 only, and there was no significant interaction with tau [29]. These findings suggested that UA had protective actions against longitudinal decline of cognition and can interact with A*β* and tau. Higher level of SUA may indicate slower progression of AD. The results also showed sex-related differences in uric acid's protective effects on cognition, which may partially explain the conflicting results and conclusions of the two cohort studies mentioned above.

This sex-related effect has also been observed in some other studies. A study of 1451 cognitively healthy adults found that elevated baseline SUA was relevant to decreased attention and visuospatial abilities in males. But in females, there were no marked findings [30]. A longitudinal cohort study of SUA and cognitive change showed that elevated level SUA at baseline was relevant to faster decline of cognition by using the visual memory/visuoconstruction ability test. UA only had a potential benefit for attention filed in older men [31]. Lin et al. found that the high level of SUA had different actions on spontaneous brain activities and cognitive function in men and women [32]. This sex-dependent effect was also suggested in a cross-sectional analysis from the ELSA Brazil cohort [33].

Some scholars have pointed out that the conclusions of these studies may be due to the different standard SUA levels of men and women. Many studies have shown that women and men have different optimal SUA cutoff values for predicting metabolic syndrome, insulin resistance, cardiovascular status, the development of gout, etc. [34, 35]. Since the cutoff value for hyperuricemia in women is lower than that in men, at a certain high uric acid level, men and women actually have different elevations. If this had been taken into account in the data analysis, different results might have been found [36]. Honarpisheh et al. [37] also pointed out that sex was a biological variable in the pathological studies of neurodegenerative diseases. In future research, this factor should be considered in data analysis.

There were some studies on the influence of SUA on cognitive function, which were not AD targeted. In a cross-sectional analysis, Xue et al. found that levels of SUA were markedly decreased in MCI patients (292.28 ± 63.71 *μ*mol/L) compared to HC (322.49 ± 78.70 *μ*mol/L; *P* < 0.05). Markedly positive correlations were shown between the Mini-mental State Examination (MMSE) scores and levels of SUA (*P* < 0.05). UA was a protective factor for MCI (odds ratio = 0.999, 95% CI = 0.987-0.999) [38]. Similar results were found in a cross-sectional study of 10,016 individuals in Beijing, China [39].

Wang et al. enrolled 12798 middle-aged and elderly people over 45 years old in the follow-up study of health and pension in China. They measured the baseline plasma UA level, and the cognitive function was evaluated by a variety of methods. The results showed that middle-aged and older Chinese with high UA levels at baseline had better cognitive function, but not with rates of cognitive decline [40]. Xu et al. found that lower serum UA levels were related to cognitive dysfunction and could serve as a potential predictor for VD [41]. In a cross-sectional study in South Korea, Kim et al. observed the association along SUA, AD brain changes, and cognitive impairment. 430 dementia-free elderly subjects were enrolled in this study. The results suggested that levels of SUA were markedly relevant to AD-characteristic area cerebral glucose metabolism (AD-CM) and borderline associated with MMSE. AD-CM was the link of UA and cognitive measure scores. A decreased level of SUA was relevant to AD-related cerebral hypometabolism [42]. In previous studies, few studies had included multimodal brain imaging to observe the relationship between uric acid and brain changes. Subsequent research is needed to prove whether this represents a causal relationship or not. And only after this relationship has been confirmed by more studies can experiments and clinical trials be designed from this point to using SUA as a therapeutic target.

In contrast, in a case-control study, higher levels of circulating UA were considered to be relevant to impairment of cognition in pharmacologically untreated elderly subjects [43]. In a two-center study of 180 elderly maintenance hemodialysis (MHD), Zhang et al. found that SUA levels were independently and negatively correlated with MMSE scores [44]. High SUA level may lead to impairment of cognition in the elderly MHD patients. A cross-sectional study from Japan showed that the high level of SUA was independently related to deterioration of cognition, and UA had an adverse influence on cognition [45].

### 4.2. Summary of This Section

Existing cohort studies have suggested that the high SUA level was associated with slower cognitive decline in AD patients, and high concentration of SUA could attenuate the effects of A*β*1-42 and tau on decline of cognition in female subjects. This gender-related effect has also been observed in some other studies, which may be due to the fact that the standard SUA level of men and women is different. A large number of cross-sectional studies found that the elderly with high UA level had better cognitive function and SUA was considered to be a protective factor of MCI. The decreased level of SUA was relevant to AD-related cerebral hypometabolism, but it is still uncertain whether there is a causal relationship. Still, some studies have shown that UA had an adverse influence on cognition (Table 1).

## 5. UA Was Related to AD through Multiple Mechanisms

Based on former research, UA had a dual effect on cognition including neuroprotective action and neurotoxic action. The dual effect of UA is reflected in a variety of hypotheses, including oxidative stress (OS), interaction with *β*-amyloid (A*β*), inflammation, endothelial dysfunction, and vascular damage [3].

Current research indicates that OS has a crucial part to play in these mechanisms. The influence of OS is particularly important in neurodegenerative diseases. The brain is especially vulnerable to reactive oxygen species (ROS). Lipids, proteins, and nucleic acids of neurons can be attacked by ROS, causing inevitable neuronal dysfunction [11]. Evidence showed that the brain tissue of AD patients is exposed to OS, which leads to peroxidation of lipid, protein, DNA, and RNA and glycoxidation [46]. These peroxidation products could promote the production of key pathological changes of AD, including A*β*, neurofibrillary tangles (NFT), and inflammation. In turn, pathological changes of AD could promote oxidative stress, which has been experimentally demonstrated in postmortem brain tissues in AD patients and AD transgenic mouse models [47]. Therefore, the two aspects interacted with each other and accelerated the development of AD. Additionally, it has been demonstrated that increasing antioxidative potential correlated with a reduction of white matter injury [42].

**Figure 2:**
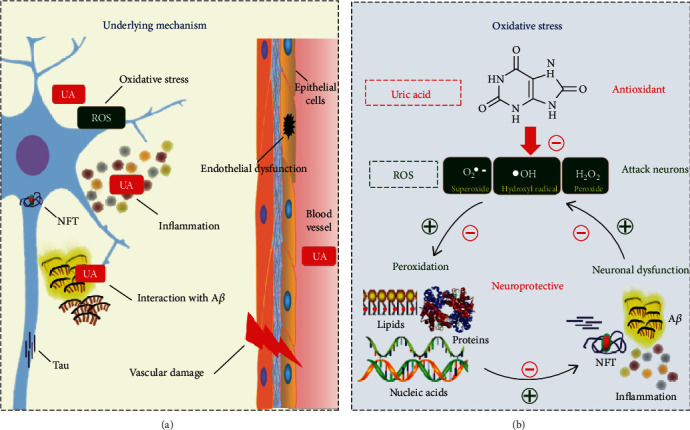
The underlying mechanism of uric acid on AD: (a) a summary figure on potential mechanisms; (b) SUA and AD were connected by oxidative stress.

UA is a natural and powerful antioxidant of humans. The antioxidant effect of UA is attributed to its capacity to chelate transition metal ions to make stable complexes [48, 49] and to act as a powerful cleaner of oxygen and hydroperoxyl radicals. The level of UA in cerebrospinal fluid was positively correlated with SUA level, especially when the blood-brain barrier was damaged [50]. Therefore, a higher serum uric acid level indicates a higher antioxidant capacity and may reduce the damage to the brain from oxidative stress. However, the antioxidant properties have also been challenged by some studies. Some scholars have suggested that under some specific conditions, uric acid even had an oxidation-promoting effect [51–53]. And there was insufficient data to analyze uric acid levels in the brain, and the limited available data did not suggest a significant change [54, 55] (Figure 2).

An in vivo experiment conducted by Shao et al. showed that UA induced hippocampal inflammation through the TLR4/NF-*κ*B pathway and led to dysfunction of cognition. They also found that hyperuricemia in rats and people was relevant to gliosis in the hippocampus by using magnetic resonance imaging [56]. Another laboratory study showed that ITM2B was a regulator of GLUT9-mediated urate transport, which was a molecular link between UA homeostasis and neurodegenerative diseases [57]. Mazumder et al. [58] pointed out that uric acid was the most potent inhibitor of AChE, which was associated with dementia and cognitive impairment. In the Framingham Study, Chouraki et al. found the association with AD and hypoxanthine and taurine, which could be evidence that UA is neuroprotective [59].

Other potential links between uric acid and cognitive impairment can be suggested from the perspective of genetic syndromes and malnutrition. Some genetic syndromes are characterized by congenital uric acid disorders and neurological deficits, for example, primary renal hypouricemia [60, 61] and Lesch-Nyhan syndrome [62]. Therefore, it can be inferred that patients with the congenital uric acid disorder may have a genetic tendency to cognitive dysfunction. Since high uric acid levels increase cardiovascular risk, the relation between uric acid level and neurologic disorders could be like U- or J-shaped [61], which means if the UA level is extremely high or low, the risk of AD may both increase.

Hypouricemia is recognized as a sign of poor nutritional status, and some studies showed that poor nutritional status can cause faster cognitive decline in people with dementia [63]. There is a possibility that the correlation between low uric acid and cognitive impairment shown in the above study may include the influence of malnutrition. Cognitive frailty is a clinical syndrome in elderly individuals, which is characterized by physiological weakness and potentially reversible cognitive impairment, and dementia is excluded. The concept describes a preclinical cognitive status caused by physical frailty rather than neurodegenerative disorders [64]. The emergence of the concept of cognitive frailty supported this point. It provided a possibility that cognitive impairment may be caused by frailty or other comorbidities, and UA level is the manifestation of malnutrition, frailty, or other comorbidities rather than the main determinant.

## 6. Conclusions

The relationship between SUA and AD remains controversial, while current evidence supports the hypothesis that elevated UA levels could reduce the risk of AD, slow down the decline of cognition, and delay the progression of AD, and high SUA level may related to lower risk and progression of AD. But findings of some studies contradicted it. The main reasons for contradiction between the results are study inconsistency, the difference in subject selection (sex and age), and the lack of subtype analysis of incident dementia.

The current studies have some shortcomings, such as only recorded the UA level at baseline, one-time record could not reflect long-term uric acid levels, the small number of large-sample, AD-targeted cohort studies, and inadequate adjustment of confounding factors like nutritional status and comorbidity.

Whether the dual effect of UA can be selectively controlled, whether it has different effects on men or women, and whether there is a causal relationship between low SUA and AD-related cerebral hypometabolism are questions that need to be considered in the design of future studies. Future epidemiological studies should measure the SUA level several times to record dynamic changes and analyze the actual increase of SUA in different genders, carry out the assessment of nutrition, frailty, and comorbidity to fully exclude the influence of confounding factors, and combine imaging examinations in the study design.

## Figures and Tables

**Table 1:** Latest prospective studies that assessed the relationship between SUA and AD.

First author, journal year [Ref]	Population	Duration	Main results
Scheepers et al. 2019 [4]	1462 females	44 years	Lower risk of AD in women with higher SUA (HR 0.78; CI 0.66–0.91).
Latourte et al. 2018 [5]	1598 individuals	12 years	Significant risk of VD or mixed dementia in patients with higher SUA levels (HR 3.66, 95% CI: 1.29–10.41, *P* = 0.015), compared to AD (HR 1.55 (95% CI 0.92 to 2.61), *P* = 0.10).
Alam et al. 2020 [6]	11,169 individuals	24.1-year median follow-up period	After adjustment including cardiovascular risk factors, SUA and incident dementia showed an uncorrelated outcome (HR, 1.03; 95% CI, 0.88, 1.21). Elevated baseline levels of SUA were relevant to faster decline of cognition (25-year global *z*-score difference, -0.149; 95% CI, -0.246, -0.052).
Cao et al. 2020 [7]	502,528 individuals	8.1-year median follow-up period	People in the lowest level group of SUA had a 25% increased risk of dementia compared to those in the highest SUA level group (HR = 0.75, 95% CI: 0.64-0.87).
Ye et al.2016 [29]	1064 subjects (197 AD, 596 MCI, and 271 HC)	Mean duration 2.9 years	Higher levels of uric acid were associated with slower cognitive decline, particularly in the MCI and AD subgroups and more prominently in female subjects (*P* < 0.01).
